# The COVID-19 pandemic preparedness simulation tool: CovidSIM

**DOI:** 10.1186/s12879-020-05566-7

**Published:** 2020-11-19

**Authors:** Kristan A. Schneider, Gideon A. Ngwa, Markus Schwehm, Linda Eichner, Martin Eichner

**Affiliations:** 1 Department of Applied Computer- and Bio-Sciences, University of Applied Sciences Mittweida, Technikumplatz 17, Mittweida, 09648 Germany; 2 Eberhard Karls Universität Tübingen, Geschwister-Scholl-Platz, Tübingen, 72074 Germany; 3 Department of Mathematics, University of Buea, Buea, Cameroon, P.O. Box 63, South West Region, Buea, Cameroon; 4 ExploSYS GmbH, Otto-Hahn-Weg 6, Leinfelden-Echterdingen, 70771 Germany; 5 Public Health Office Reutlingen, St.-Wolfgang-Str. 13, Reutlingen, 72764 Germany; 6 Epimos GmbH, Uhlandstr. 3, Dusslingen, 72144 Germany

**Keywords:** SARS-CoV-2, SEIR model, Mathematical model, Social distancing, Case isolation, Control intervention, Seasonal variation

## Abstract

**Background:**

Efficient control and management in the ongoing COVID-19 pandemic needs to carefully balance economical and realizable interventions. Simulation models can play a cardinal role in forecasting possible scenarios to sustain decision support.

**Methods:**

We present a sophisticated extension of a classical SEIR model. The simulation tool CovidSIM Version 1.0 is an openly accessible web interface to interactively conduct simulations of this model. The simulation tool is used to assess the effects of various interventions, assuming parameters that reflect the situation in Austria as an example.

**Results:**

Strict contact reduction including isolation of infected persons in quarantine wards and at home can substantially delay the peak of the epidemic. Home isolation of infected individuals effectively reduces the height of the peak. Contact reduction by social distancing, e.g., by curfews, sanitary behavior, etc. are also effective in delaying the epidemic peak.

**Conclusions:**

Contact-reducing mechanisms are efficient to delay the peak of the epidemic. They might also be effective in decreasing the peak number of infections depending on seasonal fluctuations in the transmissibility of the disease.

**Supplementary Information:**

The online version contains supplementary material available at (doi:10.1186/s12879-020-05566-7).

## Backgound

The whole world has been shaken by the outbreak of a new corona virus disease outbreak that started in Wuhan province of China in late 2019 [1]. Due to international travel and interconnectivity, the virus has spread worldwide and affected almost every major nation of the world. Though there is limited data on the extent of the spread of the infection in many parts of the world, on March 11, 2020, the WHO has declared the 2019 corona virus disease outbreak, COVID-19, a global pandemic [2]. COVID-19 is caused by a virus known as the severe acute respiratory syndrome coronavirus 2 (SARS-CoV-2) [3]. The COVID-19 outbreak, has meanwhile caused over 46 million cases and over 1.19 million deaths in over 200 countries (November 3, [4]). Human-to-human transmission can occur via droplets or contaminated surfaces and materials [5].

The symptoms of COVID-19 appear after an incubation period of 2 to 14 days (mean 5-6 days) [6, 7] and can vary in intensity, ranging from asymptomatic infections to pneumonia and subsequent death. Serious upper respiratory tract infections with case fatality rates of 1% to 5% are reported outside of mainland China, whereby the data from the Hubei province in China indicate a figure of 18% [8, 9]. Elderly and people with chronic diseases are considered a high-risk population [10]. It is however estimated that about 80% of infections only lead to mild or moderate symptoms [11–13]. The most commonly displayed symptoms are cough, fever and rhinitis [9]. In the absence of a vaccine and an approved treatment, local control of the coronavirus transmission requires a combined and coordinated control effort [4]. In an effort to decelerate the spread of this disease, many countries have taken drastic measures to reduce social contacts, for instance, by closing schools and forbidding social gatherings. Some even go as far as introducing a total system shut down. Such practices are however very costly and have devastating effects not merely on the fabric of society but also on the economy [14].

Efficient control and management can be achieved if new, more economical, and realizable methods are used. Mathematical models can play a cardinal role in forecasting possible scenarios.

This article describes the background of the COVID-19 pandemic preparedness tool, CovidSIM Version 1.0, which was made publicly available in February 2020 (http://covidsim.eu). The main purpose of CovidSIM Version 1.0, is to provide a realistic and easy-to-use simulation tool with the capacity to support decision making in public and global health, epidemiology and economy. It can be used to simulate the pandemic under different scenarios. The focus lies on establishing a model with realistic dynamics. The model is first verbally described in Methods, followed by mathematical formulations presented in Supplement file 1 which may be skipped by readers who are predominantly interested in model applications.

The dynamics of the model can be readily employed to derive variables, which are epidemiologically or economically relevant. Some of the derived quantities arise naturally in the model description, whereas others are obviously relevant and easily calculated from the dynamics implemented in CovidSIM version 1.0.

## Methods

**Fig. 1:**
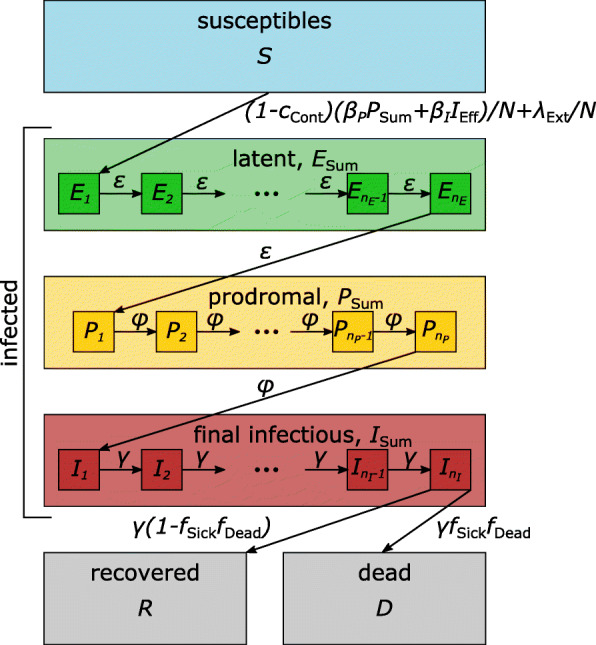
Model illustration. *I*_Eff_ is the number of contagious individuals i.e., *I*_Eff_=*I*−*I*_Iso_−*p*_Home_*I*_Home_

We model the spread of COVID-19 deterministically, using an extended SEIR model, i.e., a deterministic compartmental model of ordinary differential equations. We first describe the model verbally and provide a concise mathematical description in Supplement file 1. Figure 1 illustrates the model.

A population of size *N* is divided into susceptible, infected, and recovered individuals. During the course of the infection, individuals pass through (i) the latency period, during which they are not yet infective, (ii) the prodromal period, during which they are already infective, but the infection is still in an early state where it cannot be yet transmitted as easily as in the later period, in which viral load already increased (in this period, individuals do not yet show typical symptoms), and (iii) the final infectious period during which infected individuals may or may not have symptoms. Finally, individuals recover and obtain a full immunity or die. The model follows the time change of the number of individuals being susceptible (*S*), in the latent period (*E*), in the prodromal period (*P*), in the final infectious period (*I*), and in the final recovered (*R*) and dead (*D*) stages.

The classical approach to model the time change of the number of latent, prodromal and infected individuals would be to assume that individuals simply proceed from one stage to the next. This, however, is too simplistic, because it implies that the time-delay would be exponentially distributed, which does not appropriately describe the dynamics. To mitigate this issue, the latent, prodromal, and final infectious periods are divided into several sub-stages. Infected individuals first pass through the latent, then through the prodromal, and through the final infectious period in a stepwise process, which leads to much more realistic delays for people passing on from the latent to the prodromal period, to the final infectious period and finally to the immune stage.

As the model is built for an epidemic which occurs in a large population in a relatively short time period, deaths, which are unrelated to the disease, are ignored. Contacts between individuals are assumed to occur at random. Susceptible individuals acquire infections through contacts with individuals in the prodromal or the final infectious periods at rates *βP* and *βI*, respectively. The basic reproduction number *R*_0_ is the average number of infections caused by an infected individual in a completely susceptible population, in which no interventions occur, during the entire infectious period, consisting of the prodromal period and the final infectious period. This number summarizes all the infections, which are caused during the entire infectious period. This definition of *R*_0_ specifically requires that initially everybody but the infected individuals in the population is susceptible and there are no intervention measures. The basic reproduction number is allowed to fluctuate seasonally.

Infected individuals first become latent carriers. After that, they enter the prodromal period, in which they can infect others even before entering the final infectious period. At the beginning of the final infectious period, it is determined whether the infection proceeds as symptomatic or asymptomatic. A fraction *f*_Sick_ of individuals in the final infectious period develops symptoms. CovidSIM Version 1.0 allows to isolate individuals with symptoms and to restrict this intervention to a time interval. The detected fraction of symptomatic infections is isolated in quarantine wards, but if it is full, they go directly into home isolation. Home isolation only prevents a fraction of contacts, while quarantine wards are assumed to prevent all infectious contacts.

After the final infectious period, symptomatic infections result in death with a given probability. Infected individuals that do not die at the end of the final infectious period become permanently immune.

Furthermore, CovidSIM allows addressing time-dependent interventions to prevent contacts because of social distancing measures.

### Derived quantities

In this section, the derived quantities used in CovidSIM are described verbally; a mathematical description is provided in Supplement file 2.

A fraction of infected individuals develops symptoms in the final infectious period, while the rest remains asymptomatic. We first calculate the number of symptomatic infections and then multiply this number with fractions in order to calculate the demand for medical care, hospital capacities, isolation facilities, and intensive care units (ICU), respectively. These derived quantities only provide additional output of CovidSIM, but they do not influence the model dynamics.

The cumulative disease incidence is a measure which is frequently reported. This is given by the number of cases that have occurred since the start of the outbreak. The number of symptomatic infections occurring within a given time interval is obtained by cumulating the fraction of all infections that progress from the prodromal period to the final infectious period and become symptomatic. Infections are cumulated over the appropriate time interval to obtain the daily and weekly numbers of cases, hospitalizations, etc. They are referred to as daily and weekly incidences, respectively.

### Implementation of CovidSIM

The web interface of CovidSim Version 1.0 is implemented in Javascript. The source code is available on GitLab under https://gitlab.com/exploratory-systems/covidsim/. CovidSIM uses an ODE solver to run the simulation in the browser of the clients, based on a fourth-order Runge-Kutta method with step size control as described in [15]. The original Fortran code was translated into Java code and JavaScriptCore, V8 using the Web Workers API to facilitate a series of simulation requests in parallel on the host computer’s CPUs. The web application is created in typescript with Angular, ngrx, rxjs, and observable-webworker. The user interface uses Angular Material, d3, ng2-dvd3.

### Parameter choices

Model parameters as they occur in the web interface are summarized in Table 1 with their corresponding notation in the model description. By default, at the start of the simulation, one infection is introduced in a fully susceptible population of 100 million inhabitants. Furthermore, one infection per day originating from outside the population is assumed. The number of initial infections (1-1,000 infections at the start of the simulation), the size of the population (1-1,000 million) and the number of infections from outside (1-100) per day can be adjusted by the user. Other parameters such as timing and duration of preventative measures such as social distancing can also easily be adjusted by the user.

**Table 1:** Web interface and model parameters of CovidSIM Version 1.0

Parameter name on the CovidSIM interface	Parameter	Unit	Default value
Population:			
Population size	*N*	million	100
Number of initial infections	*L*_Init_	individuals	1
Infections from outside of the population	*λ*_Ext_	per day	1
Durations:			
Latency period average duration	*D*_*E*_=*n*_*E*_/*ε*	days	4
Number of latency stages	*n*_*E*_		16
Prodromal period average duration	*D*_*P*_=*n*_*P*_/*φ*	days	1
Number of prodromal stages	*n*_*P*_		16
Final infectious period average duration	*D*_*I*_=*n*_*I*_/*γ*	days	10
Number of final infectious stages	*n*_*I*_		16
Severity:			
Infections which will lead to sickness	*f*_Sick_	%	58
Sick patients seek medical help	*f*_Consult_	%	40
Sick patients are hospitalized	*f*_Hosp_	%	2
Hospitalized cases need intensive care	*f*_ICU_	%	2
Sick patients die from the disease	*f*_Dead_	%	2
Contagiousness:			
Annual average of the basic reproduction number	$\bar R_{0}$		4
Amplitude of the seasonal fluctuation of *R*_0_	*a*	% of $\bar R_{0}$	0
Day when the seasonal *R*_0_ reaches its maximum	$t_{\mathrm {R}_{0_{\max }}}$	day	0
Relative contagiousness in prodromal period	*c*_*P*_	%	50
Interventions:			
General contact reduction	*p*_Dist_(*t*)	%	50
General contact reduction begin	$\phantom {\dot {i}\!}t_{\text {Dist}_1}$	day of	0
General contact reduction duration	$\phantom {\dot {i}\!}t_{\text {Dist}_2}-t_{\text {Dist}_1}$	day	0
Probability that a sick patient is isolated	*f*_Iso_	%	0
Maximum capacity of isolation units	*Q*_max_	abs. number	0
Contact reduction for cases in home isolation	*p*_Home_	%	0
Begin of case isolation measures	$\phantom {\dot {i}\!}t_{\text {Iso}_1}$	day	0
Duration of case isolation measures	$\phantom {\dot {i}\!}t_{\text {Iso}_2}-t_{\text {Iso}_1}$	day	0

## Results

To exemplify the dynamics of the COVID-19 pandemic, parameters are adjusted to reflect the situation in the Federal Republic of Austria, one of the first countries in Europe that implemented strict control interventions. Parameter values are listed in Tables 2 and 3, which follow literature values (cf. [9]) or data regularly published by the John Hopkins University (https://coronavirus.jhu.edu/data).

**Table 2:** Parameter values used in Figs. 2, 3, 4, 5 and 6. Note, *a*=0 implies no seasonal variation

	parameter	value
Population	*N*	9 million
	*I*(0)	15
	*λ*_Ext_	10/day
Durations	*D*_*E*_=*n*_*E*_/*ε*	4 days
	*n*_*E*_	16
	*D*_*P*_=*n*_*P*_/*φ*	1 day
	*n*_*P*_	16
	*D*_*I*_=*n*_*I*_/*γ*	10 days
	*n*_*I*_	16
Severity	*f*_Sick_	82%
	*f*_Dead_	7
Contagiousness	$\bar R_{0}$	4
	*a*	0% or 43%
	$t_{\mathrm {R}_{0_{\max }}}$	day 290
	*c*_*P*_	50%
Interventions	$\phantom {\dot {i}\!}t_{\text {Iso}_1}$	day 30
	$\phantom {\dot {i}\!}t_{\text {Iso}_2}-t_{\text {Iso}_1}$	365 days

**Table 3:** Different parameters used in Figs. 2, 3, 4, 5 and 6. The table lists the specific parameter choices for the figures. Parameters which are not modified are marked with ‘-’

	Figures					
parameter	2	3&4a-c	3&4d-f	5&6a-c	5&6d-f	5&6g-i
*p*_Dist_(*t*)	75%	0-80%	75%	0%	0%	0%
*f*_Iso_	0 or 66%	0%	0%	0-80%	66%	66%
*Q*_max_	200/10,000	-	-	200/10,000	0-200/10,000	200/10,000
*p*_Home_	0 or 75%	-	-	0%	0%	0-80%
$\phantom {\dot {i}\!}t_{\text {Dist}_2}-t_{\text {Dist}_1}$	30 days	0 days	0-56 days	-	-	-

It was assumed that the first cases were introduced in the middle of February 2020, so that *t*=0 corresponds to that day of introduction. As a baseline, a constant *R*_0_=4 was assumed. To study the effects of seasonal variation in *R*_0_, a yearly average $\bar R_{0}=4$ was assumed with a 43% fluctuation during the year and a peak in late December ($t_{\mathrm {R}_{0_{\max }}}=200$). The average latency period *D*_*E*_ was assumed to be 4 days, the prodromal period (*D*_*P*_) 1 day, and the infective period 10 days (*D*_*I*_). In the prodromal period, individuals were supposed to be half as contagious as in the final infectious period.

**Fig. 2:**
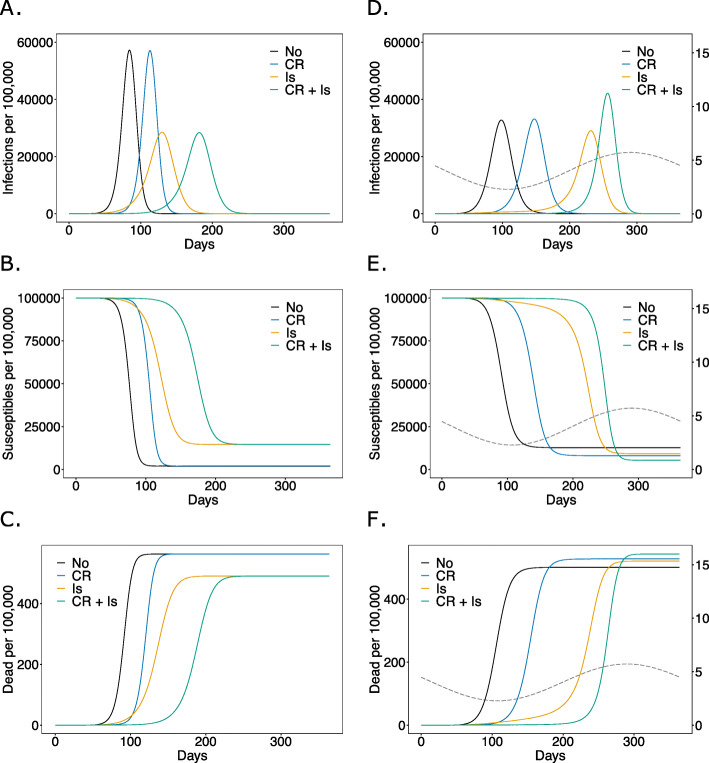
Dynamics for different interventions. Panels (**a**)-(**c**) assume no seasonal fluctuations in *R*_0_, while panels (**d**)-(**f**) assume seasonal fluctuations. The dashed lines in (**d**)-(**f**) shows *R*_0_(*t*) corresponding to the *y*-axis on the right. Panels (**a**) and (**d**) show the numbers of infected individuals, *I*(*t*), (**b**) and (**e**) the number of susceptible individuals *S*(*t*) and (**c**) and (**f**) the number of dead individuals. Each panels shows the dynamics under four different scenarios: no intervention (No); general contact reduction of 75% for 30 days, starting at day 30 (CR); isolation of symptomatic infections in quarantine wards (assuming a capacity of 200 per 10,000) or at home, assuming a contact reduction of 75% (Is), which start at day 30 and are sustained throughout the end of the simulation; contact reduction and isolation combined (CR+Is). The parameters used are listed in Tables 2 and 3

The impact of general contact reductions and case-isolation are depicted in Fig. 2. The various aspects of both interventions are explained below. Seasonal variation has an impact on both the location and the height of the epidemic peak.

### No interventions

The case without interventions needs to be understood as a reference scenario. It leads to a peak of infections which occurs approximately 90 days after the first infection.

### General contact reduction – social distancing

**Fig. 3:**
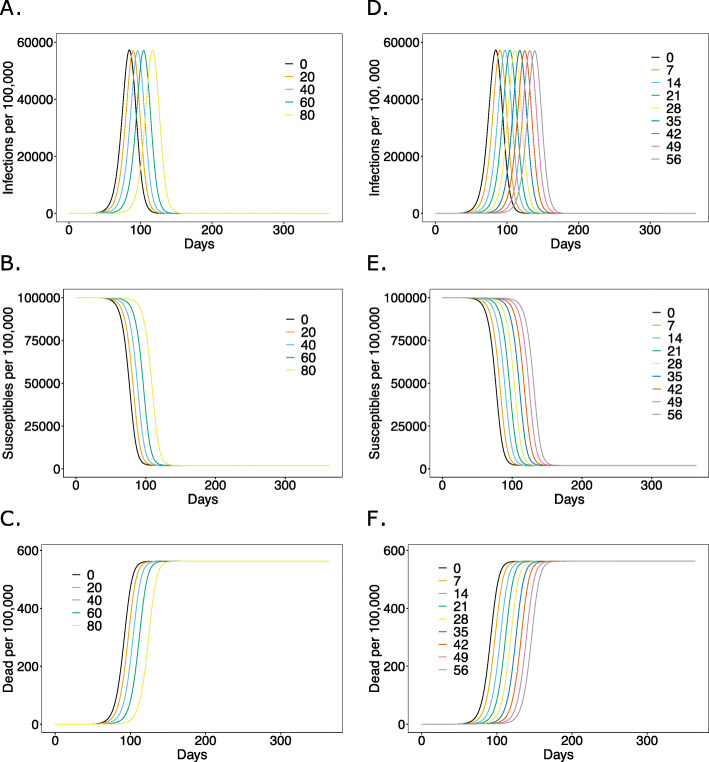
Effect of social distancing. The panels show the number of infected (*I*), susceptible (*S*), and dead (*D*) individuals at time *t*, respectively, for different effectiveness (**a**-**c**) and durations (**d**-**f**) of general contact reducing by social distancing. All figures assume that cases are not isolated. Panels (**a**)-(**c**) show the effect of a 30-day of 0-80% contact reduction starting at day 30. Panels (**d**)-(**f**) show the effect of 75% contact reduction starting on day 30 and lasting for different time periods. The parameters used are listed in Tables 2 and 3

**Fig. 4:**
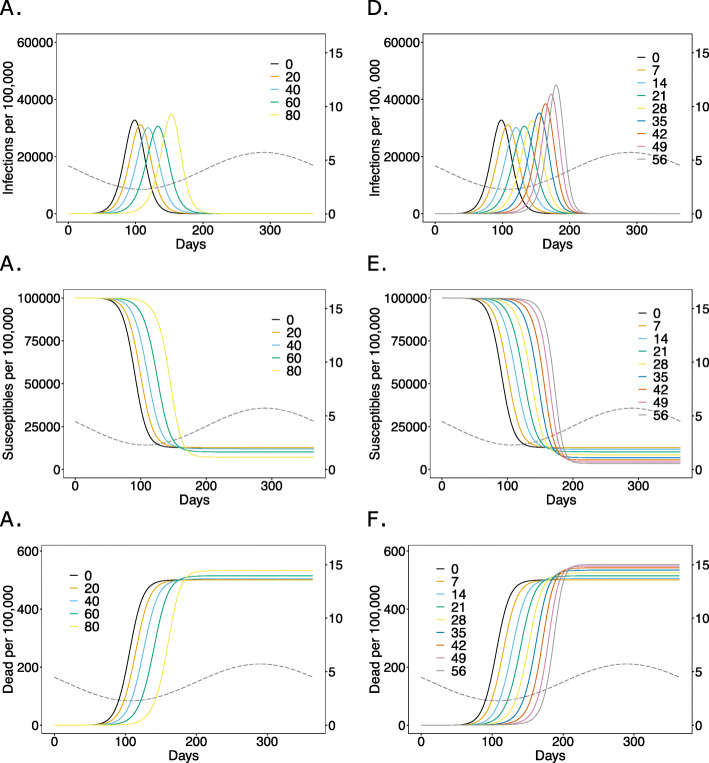
Effect of general contact reduction under seasonal fluctuations. As Fig. 3, but assuming seasonal fluctuations in *R*_0_. The dashed line shows *R*_0_(*t*) corresponding to the *y*-axis on the right

Assuming a contact-reducing intervention (social distancing) of 30 days, starting at day 30, the measures delay the peak (Figs. 3 and 4). The more efficient the reduction of person-to-person contacts, the longer is the delay.

In the absence of seasonal fluctuations of *R*_0_, social distancing leads only to a delay of the epidemic peak but not to a reduction of its height. In particular, the number of deaths throughout the pandemic is independent of such interventions. More severe contact reductions lead to a disproportionally long delay (Fig. 3a-c).

The peak’s height is affected by seasonal fluctuations and becomes lower or higher due to the timing of the fluctuations (Fig. 4a-c). Importantly any delay will – in the absence of any other control measures – ultimately result in a higher number of deaths in the population if the peak is shifted into a season with higher *R*_0_.

Assuming a general reduction of 75% of all contacts, the duration of the interventions scale linearly with the shift of the epidemic peak (Fig. 3d-f), yet the height of the peak is unaffected if there is no seasonality. This changes with seasonal fluctuations, where the peak’s height can substantially increase in the flu season, resulting in higher mortality (Fig. 4d-f).

Importantly, effectiveness and duration of general contact-reducing measures show bigger delays in the case of seasonal fluctuations if they are implemented during a time in which *R*_0_ is low.

### Isolation of symptomatic cases

**Fig. 5:**
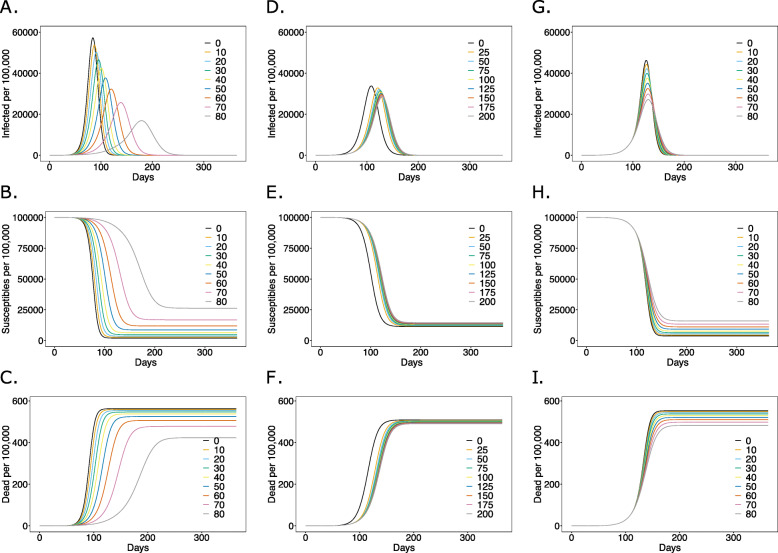
Effect of isolating cases. The panels show the number of infected (*I*), susceptible (*S*), and dead (*D*) individuals at time *t*, respectively, for different percentages of symptomatic cases being isolated (**a**-**c**), isolation ward capacity per 10,000 inhabitants (**d**-**f**) and percentage of contact reduction in home isolation (**g**-**i**). The case-isolation measures are implemented on day 30 and last until the end of the simulation. No general contact-reducing measures are assumed. Panels (**a**)-(**c**) assume that home isolation reduces 75% of the contacts of the isolated cases; the capacity of quarantine wards is set to 200 per 10,000; the percentages of symptomatic cases which are isolated are shown by different colors. Panels (**d**)-(**f**) assume that 66% of symptomatic cases are detected and isolated; home isolation prevents 75% of contacts; the capacities of quarantine wards per 10,000 are shown by different colors. Panels (**h**)-(**i**) assume a capacity of quarantine wards of 200 per 10,000; 66% of symptomatic cases are detected and isolated; percentages of contact reductions in home isolation are displayed in different colors. The parameters used are listed in Tables 2 and 3

**Fig. 6:**
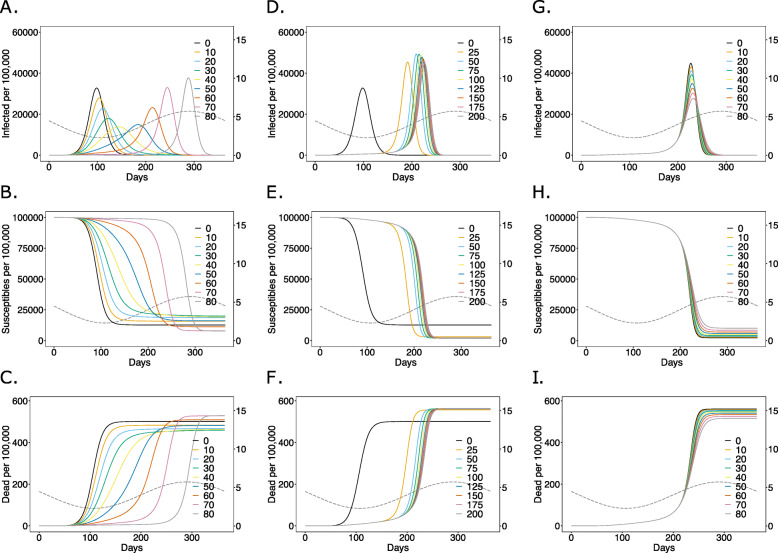
Effect of isolating symptomatic cases when considering seasonal fluctuations. As Fig. 5, but assuming seasonal fluctuations in *R*_0_. The dashed lines shows *R*_0_(*t*) which is displayed on the right vertical axis

Isolating cases has a profound effect on the location and the height of the peak. Assuming that no further person-to-person contact reductions take place and that there is a capacity of 200 isolation wards per 10,000 individuals, and a reduction of contacts by 75% in home isolated cases delays the peak substantially (Figs. 5a-c and 6a-c).

The shift in the peak does not scale linearly, but is disproportionally long for high percentages of infections being isolated. Additionally, in the absence of seasonal fluctuations, the peak will decrease profoundly, resulting in reduced mortality (Fig. 5a-c). With seasonal fluctuations in *R*_0_, the peak’s shift is more pronounced if *R*_0_ originally is seasonally low. However, it starts to increase, if the delay shifts the peak into the season with higher transmissibility (Fig. 6a-c). Notably, it is assumed that the isolation measures are continued until the end of the simulation time. Once these are discontinued, some of the susceptible individuals will get infected.

Assuming that 66% of symptomatic infections are isolated and a reduction of 75% of contacts in home isolation, we studied the effect of the capacity of isolation wards. An increase in the capacity of wards shifts and decreases the peak (Fig. 5d-e). However, the gain of further increasing the wards’ capacities in shifting and decreasing the peak soon become insignificant. Seasonal fluctuations in *R*_0_ again complicate the picture. While the shift in the peak is more pronounced if *R*_0_ initially is low, the height of the peak increases or decreases, depending on its timing (Fig. 6d-e).

Finally, assuming (a) an isolation capacity of 200 wards per 10,000 individuals and (b) 66% of the symptomatic infections being isolated, and (c) decreasing the contact rate of home isolated individuals, does not significantly delay the peak, but reduces its height (Figs. 5g-i and 6g-i). This is due to the effective reduction of infectious contacts. This effect is hardly affected by seasonal variations in *R*_0_ because it has only a minor delaying effect.

### Combination

In the absence of seasonal fluctuations, combinations of both isolation mechanisms result in the longest delay and in the best reduction of the peak’s height (75% reduction of contacts in home isolation, a fraction of 66% of symptomatic infections being isolated, isolation ward capacity of 200 places per 10,000 individuals, 75% general reduction of contacts for 30 days starting at day 30). Notably the delaying effect is mainly due to the solation of cases, not to social distancing measures. The height of the epidemic peak is clearly affected by seasonal fluctuations in *R*_0_ (compare Fig. 2a-c with d-f). In the absence of seasonal fluctuation, general contact reduction measures have just a shifting effect on the peak (because the intervention is limited in time), whereas the isolation of sick individuals has a shifting and reducing effect on the peak (because the intervention is not limited in time). Seasonal fluctuation affects both the extent of the delay and the height of the peak in a pattern that can better be understood when looking at the seasonal values of *R*_0_.

## Discussion

The results suggest that temporal social distancing measures will simply shift the epidemic peak essential by the time the interventions are sustained. If there is seasonal variation, the peak can be shifted towards the flu season, which ultimately leads to increased morality. The isolation of sick cases is more efficient to delay the epidemic peak and to lower it. However, such a strategy results in a higher number of cases and thus higher mortality, if the resulting delay shifts the peak into a period with higher transmissibility. A combination of both measures is most efficient in delaying the epidemic peak and keeping the number of infections and COVID-19-related deaths low. However, seasonal fluctuations can again cause higher mortality. Such a combined strategy is, thus, only meaningful if there is confidence to rapidly develop better treatments for symptomatic cases, prophylaxes, or immunizing vaccines, or if more time is necessary to prepare the healthcare systems to face a full pandemic outbreak. It should be mentioned, that the considerations here are purely epidemiologically. Evolutionary considerations should also be taken into account. Namely, it is unclear to what extent the virus can mutate during the pandemic.

Importantly, general social-distancing interventions cannot simply be decided, as they are subject to individual behavioral decisions. The model clearly assumes that social distancing is temporary and the contacts will reach normal levels again as soon as this intervention has been abolished. This, however, seems hardly plausible, as people will most likely stick to contact-avoiding behaviors in the aftermath of such interventions. Yet, there is no guarantee that this kind of interventions can be sustained over extended periods of time. In particular, the achievable reduction in contact behavior depends on the economic situation, the available infrastructure and cultural habits. Also, the effect of home isolation has to be treated with caution, as it assumes that disease-negative individuals do not constrain their contacts. This again seems implausible as many COVID-19-free individuals may also retreat into self-imposed isolation if they experience influenza-like symptoms. Again, the effectiveness of home isolation depends on several factors as the economic status of a society, its hygienic standards, the family size distribution etc.

Notably, our predictions essentially assume a closed population which is exposed to a few infections imported from outside (which do not strongly affect the dynamics once the infection starts to spread in the population). However, in the absence of restrictions in mobility and international travel, a population cannot be considered as independent. Effective social distancing essentially assumes that hardly any infection is imported, thus, in essence that boarders are closed. Nevertheless, CovidSIM is a convenient tool to get robust estimates of the impact of several interventions and thereby facilitates decision-support. However, other confounding factors including ethical, cultural and economic ones need to be considered and the interpretation of predictive results should be discussed with experts in the field of infectious disease modeling.

The numbers of symptomatic infections, hospital or ICU capacities, and mortality are derived quantities that can be deduced in an age-dependent fashion from the model output. However, CovidSIM Version 1.0 does not yet explicitly incorporate age structure, and particularly age-dependent contact behaviour, in the model. Thus, CovidSIM Version 1.0 is more relevant for industrialized nations than for countries in which the demography is strongly dominated by an expanding population, e.g., for many countries in Subsaharan Africa. where adjustments to the model would be necessary. Future extensions of CovidSIM, building upon the basic model described here, will include age structure explicitly.

## Concluions

CovidSIM allows predictions on the COVID-19 pandemic under various interventions. The tool will be extended in the future by necessary and meaningful model refinements and additional interventions which are emerging as control measures are being discussed and implemented. All planned extensions will be based on the model described here. By using substages, the model avoids exponentially distributed durations of disease stages that emerge implicitly from the classical SEIR type models.

The CovidSim web interface was employed to explore the impact of different control measures with parameter choices that roughly reflect the situation in the Federal Republic of Austria as an example. The purpose was to illustrate the effects of different control strategies and their combined effects. Any decision based on these results must be taken with caution, especially attempts to optimally control the disease.

## Supplementary Information

**Additional file 1** Mathematical description of the model

**Additional file 2** Mathematical description of derived quantities

## Data Availability

All data has been generated with CovidSIM Version 1.0. available at http://covidsim.eu. The source code is available at GitHub (https://gitlab.com/exploratory-systems/covidsim/).

## Abbreviations

SEIR: Susceptible, exposed, infected, recovered
WHO: World health organisation
COVID-19: Coronavirus disease 2019
SARS-CoV-2: Severe acute respiratory syndrome coronavirus 2
ICU: Intensive care units
API: Application programming interface
