# Supplementary material for: The COVID-19 pandemic preparedness simulation tool: CovidSIM
Source: BMC Infect Dis. 2020 Nov 19;20:859. doi: 10.1186/s12879-020-05566-7 (PMC7675392; doi:10.1186/s12879-020-05566-7)
Supplement: Supplementary file 1 — Additional file 1 Mathematical description of the model [file 12879_2020_5566_MOESM1_ESM.pdf]

Additional Files

Additional file 1 — Mathematical description of the model

A population of size N individuals is assumed. The model follows the time change of the number of individuals being susceptible (S), in the latent period (E_{Sum}) consisting of n_E sub-states ($E_k,\ k = 1, \ldots, n_E$), in the prodromal period (P_{Sum}) consisting of n_P substates ($P_k,\ k = 1, \ldots, n_P$), or in the final infectious period (I_{Sum}) consisting of n_I substates ($I_k,\ k = 1, \ldots, n_I$), in the final "removed" stage (R), or dead individuals (D). Therefore, the numbers of individuals in the latent, prodromal, and final infectious periods are given by

$$E_{\text{Sum}}(t) = \sum_{k=1}^{n_E} E_k(t), \tag{1a}$$

$$P_{\text{Sum}}(t) = \sum_{k=1}^{n_P} P_k(t), \tag{1b}$$

and

$$I_{\text{Sum}}(t) = \sum_{k=1}^{n_I} I_k(t), \tag{1c}$$

A fraction f_{Sick} of individuals in the final infectious period has a symptomatic infection, i.e., they get sick, resulting in

$$I_{\text{Sick}}(t) = f_{\text{Sick}} \sum_{k=1}^{n_I} I_k(t). \tag{2}$$

such cases. A fraction f_{Iso} of symptomatic infections get hospitalized, and will be put into isolation wards, until its maximum capacity ($Q_{\max}$) is reached, in which case they go into home isolation. Isolation measures are sustained only in the time interval from t_{Iso_1} to t_{Iso_2}. Thus, the number of individuals in quarantine wards and home isolations at time t are, respectively,

$$I_{\text{Iso}}(t) = \min\left\{Q_{\max}, f_{\text{Iso}} I_{\text{Sick}}(t)\right\} \mathbb{1}_{[t_{\text{Iso}_1}, t_{\text{Iso}_2}]}(t) \tag{3}$$

and

$$I_{\text{Home}}(t) = \max\{0, f_{\text{Iso}} I_{\text{Sick}}(t) - Q_{\max}\}\big)\, \mathbb{1}_{[t_{\text{Iso}_1}, t_{\text{Iso}_2}]}(t). \tag{4}$$

where, $\mathbb{1}_A(t)$ denotes the indicator function, attaining the value 1 if $t \in A$ and 0 if $t \notin A$. Quarantine wards are supposed to prevent all contacts, whereas home isolation only prevents a fraction p_{Home}. Thus, the number of individuals in the final infectious period at time t that can infect susceptible individuals is effectively reduced to

$$I_{\text{Eff}}(t) = I_{\text{Sum}}(t) - I_{\text{Iso}}(t) - p_{\text{Home}} I_{\text{Home}}(t). \tag{5}$$

Succeeding the last stage of the final infectious period, symptomatic infections result in death with probability f_{Dead}, such that the number of deaths at time t increases by

$$\gamma f_{\mathrm{Dead}} f_{\mathrm{Sick}} I_{n_I}(t). \tag{6}$$

Individuals who do not die, recover from the disease and are assumed to be immune.

The average durations of the latent, prodromal and final infectious periods are denoted by D_E, D_P and D_I, respectively. The classical approach to model the time change of the number of latent, prodromal and infected individuals is to assume that individuals proceed from one state to the next at rates $1/D_E$, $1/D_P$ and $1/D_I$, respectively, leading for instance to the following differential equation for P

$$\frac{dP}{dt} = \frac{1}{D_E}E - \frac{1}{D_P}P.$$

Such an approach is, however, too simplistic because the time-delay from, e.g., the latent to the prodromal stage would be exponentially distributed, which would not appropriately describe the dynamics.

This issue is resolved here, because the latent, prodromal and final infectious periods are divided into several sub-stages. Infected individuals successively pass through n_E latent, then through n_P prodromal, and through n_I final infectious states. Individuals leave each of the latent sub-states at the rate ε with

$$\varepsilon = \frac{n_E}{D_E}, \tag{7a}$$

i.e., the average duration spent in each of the n_E prodromal sub-stages is D_E/n_E.

Similarly, all prodromal and latent sub-states have the same rates denoted by φ and γ, respectively, defined by

$$\varphi = \frac{n_P}{D_P} \quad \text{and} \quad \gamma = \frac{n_I}{D_I}. \tag{7b}$$

The basic reproduction number R_0 is the average number of infections caused by an infected individual in a completely susceptible population in which no interventions occur, during the entire period of that infected individual's infectiousness. This number summarizes all the infections, which are caused during the entire infectious period $D_P + D_I$. This definition of R_0 specifically requires that initially everybody but the infectious individual in the population is susceptible and there are no intervention measures. R_0 is assumed to fluctuate seasonally, with a peak in the winter

$$R_0(t) = \bar{R}_0\Big(1 + a\cos\Big(2\pi\frac{t - t_{\mathrm{R}_{0_{\max}}}}{365}\Big)\Big), \tag{8}$$

which is a periodic fluctuation across the year where the amplitude a $(0 \leq a \leq 1)$ mediates the seasonal effect on transmission and $t_{\mathrm{R}_{0_{\max}}}$ indicates the time at which the seasonal peak of infections is attained. $\bar{R}_0$ is the annual average of the basic

reproduction number. Correctly interpreted, $R_0(t)$ is the basic reproductive number if the disease is introduced at time t. The contact rates of susceptible individuals with prodromal or the individuals in the final infectious period are $\beta_P(t)P(t)$ and $\beta_I(t)I_{\text{Eff}}(t)$, respectively, if no interventions that further reduce contacts are performed. During the time in which the individual is in the prodromal period, he or she is not yet fully infectious. The relative infectiousness in that period is denoted by c_P, so that $\beta_P(t) = \frac{c_P R_0(t)}{c_P D_P + D_I}$, which is time-dependent as R_0 may fluctuate seasonally. Hence,

$$\beta_P(t) = \frac{c_P \bar{R}_0}{c_P D_P + D_I}\left(1 + a\cos\left(2\pi \frac{t - t_{\text{R}_{0_{\max}}}}{365}\right)\right). \tag{9}$$

Following the same reasoning, the effective contact rate of full-contagious individuals is

$$\beta_I(t) = \frac{\bar{R}_0}{c_P D_P + D_I}\left(1 + a\cos\left(2\pi \frac{t - t_{\text{R}_{0_{\max}}}}{365}\right)\right). \tag{10}$$

Finally, infections may also occur from outside of the population at a rate λ_{Ext}, which is assumed to be constant. CovidSIM also allows addressing time-dependent interventions to reduce contacts by social distancing. $p_{\text{Dist}}(t)$ denotes the fraction of contacts that are prevented by control measures. Hence, the effective contact rates, $\beta_P(t)P(t)$ and $\beta_I(t)I_{\text{Eff}}(t)$, are multiplied by $1 - p_{\text{Dist}}(t)$ while these interventions are activated. It is assumed that these contact-reducing interventions prevail only during the time interval from t_{Dist_1} tot_{Dist_2}, i.e.,

$$p_{\text{Dist}}(t) = c\,\mathbb{1}_{[t_{\text{Dist}_1}, t_{\text{Dist}_2}]}(t), \tag{11}$$

where c is the fraction of contacts that are reduced due to social distancing between time t_{Dist_1} and t_{Dist_2}.

Putting together the assumptions listed above, we have the system

$$\frac{dS}{dt} = -\left(\big(1 - p_{\text{Dist}}(t)\big)\big(\beta_P(t)P_{\text{Sum}}(t) + \beta_I(t)I_{\text{Eff}}(t)\big) + \lambda_{\text{Ext}}\right)\frac{S(t)}{N}, \tag{12a}$$

$$\frac{dE_1}{dt} = \left(\big(1 - p_{\text{Dist}}(t)\big)\big(\beta_P(t)P_{\text{Sum}}(t) + \beta_I(t)I_{\text{Eff}}(t)\big) + \lambda_{\text{Ext}}\right)\frac{S(t)}{N} - \varepsilon E_1, \tag{12b}$$

$$\frac{dE_k}{dt} = \varepsilon E_{k-1}(t) - \varepsilon E_k(t) \quad \text{for} \quad 2 \le k \le n_E, \tag{12c}$$

$$\frac{dP_1}{dt} = \varepsilon E_{n_E}(t) - \varphi P_1(t), \tag{12d}$$

$$\frac{dP_k}{dt} = \varphi P_{k-1}(t) - \varphi P_k(t) \quad \text{for} \quad 2 \le k \le n_P, \tag{12e}$$

$$\frac{dI_1}{dt} = \varphi P_{n_P}(t) - \gamma I_1(t), \tag{12f}$$

$$\frac{dI_k}{dt} = \gamma I_{k-1}(t) - \gamma I_k(t) \quad \text{for} \quad 2 \le k \le n_I, \tag{12g}$$

$$\frac{dR}{dt} = \gamma(1 - f_{\text{Dead}} f_{\text{Sick}}) I_{n_I}(t), \tag{12h}$$

and

$$\frac{dD}{dt} = \gamma f_{\text{Dead}} f_{\text{Sick}} I_{n_I}(t). \tag{12i}$$
