# Supplementary material for: The COVID-19 pandemic preparedness simulation tool: CovidSIM
Source: BMC Infect Dis. 2020 Nov 19;20:859. doi: 10.1186/s12879-020-05566-7 (PMC7675392; doi:10.1186/s12879-020-05566-7)
Supplement: Supplementary file 2 — Additional file 2 Mathematical description of derived quantities [file 12879_2020_5566_MOESM2_ESM.pdf]

Additional file 2 — Mathematical description of derived quantities

The number of symptomatic infections at time t, $I_{\text{Sick}}(t)$, are given in equation (**??**). Similarly, the number of asymptomatic cases is given by

$$I_{\text{Asympt}}(t) = (1 - f_{\text{Sick}}) \sum_{k=1}^{n_I} I_k(t). \tag{1}$$

The number of cases in quarantine wards and home isolation are given by equations (**??**) and (**??**), respectively.

For public-health management, the numbers of cases that lead to medical consultations or to hospitalization are important. CovidSIM assumes that a fraction f_{MC} of individuals in the final infectious period with symptomatic infections seeks medical help. The number of individuals at time t seeking medical help is, thus,

$$I_{\text{MC}}(t) = f_{\text{MC}} f_{\text{Sick}} \sum_{k=1}^{n_I} I_k(t). \tag{2}$$

Similarly, a fraction f_{Hosp} of symptomatic cases are hospitalized, so that their total number at time t is

$$I_{\text{Hosp}}(t) = f_{\text{Hosp}} f_{\text{Sick}} \sum_{k=1}^{n_I} I_k(t). \tag{3}$$

Furthermore, a fraction f_{ICU} of hospitalized individuals need treatment at ICUs, so that the number of these patients is

$$I_{\text{ICU}}(t) = f_{\text{ICU}} f_{\text{Hosp}} f_{\text{Sick}} \sum_{k=1}^{n_I} I_k(t). \tag{4}$$

The cumulative number of new infections occurring in the time interval from t_1 to t_2 is

$$N_{\text{Inf}}(t_1, t_2) = \int_{t_1}^{t_2} \frac{S(t)}{N} \left(\big(1 - p_{\text{Dist}}(t)\big) \big(\beta_P(t) + \beta_I(t) I_{\text{Eff}}(t)\big) + \lambda_{\text{Ext}} \right) dt. \tag{5}$$

Similarly, the number of symptomatic infections occurring between times t_1 and t_2 is derived by

$$N_{\text{Sick}}(t_1, t_2) = \int_{t_1}^{t_2} f_{\text{Sick}} \varphi P_{n_P}(t) dt. \tag{6}$$

Following the same logic, the cumulative numbers of medical consultations or of hospitalizations and ICU admissions between time t_1 and t_2 are, respectively,

$$N_{\mathrm{Cons}}(t_1,t_2) = \int_{t_1}^{t_2} f_{\mathrm{Sick}} f_{\mathrm{Consult}} \varphi P_{n_P}(t) dt, \tag{7}$$

$$N_{\mathrm{Hosp}}(t_1,t_2) = \int_{t_1}^{t_2} f_{\mathrm{Sick}} f_{\mathrm{Hosp}} \varphi P_{n_P}(t) dt, \tag{8}$$

and

$$N_{\mathrm{ICU}}(t_1,t_2) = \int_{t_1}^{t_2} f_{\mathrm{Sick}} f_{\mathrm{Hosp}} f_{\mathrm{ICU}} \varphi P_{n_P}(t) dt. \tag{9}$$

Finally, the number of deaths occurring in the time interval $[t_1, t_2]$ is

$$N_{\mathrm{Death}}(t_1,t_2) = \int_{t_1}^{t_2} f_{\mathrm{Sick}} f_{\mathrm{Dead}} \gamma I_{n_I}(t) dt. \tag{10}$$
